# Imeglimin may improve albuminuria associated with partial suppression of inflammatory and fibrotic markers in diabetic kidney disease

**DOI:** 10.3389/fphar.2026.1895272

**Published:** 2026-09-15

**Authors:** Yoshimi Muta, Hisashi Yokomizo, Takako Kawanami, Yuriko Hamaguchi, Dai Nagata, Mayuko Yamamoto, Yuki Senda, Yuichi Takashi, Shohta Kodama, Daiji Kawanami

**Affiliations:** 1 Department of Endocrinology and Diabetes, Fukuoka University School of Medicine, Fukuoka, Japan; 2 Department of Regenerative Medicine & Transplantation, Faculty of Medicine, Fukuoka University, Fukuoka, Japan

**Keywords:** diabetes, diabetic kidney disease, fibrosis, imeglimin, inflammation, oxidative stress

## Abstract

Imeglimin is an oral hypoglycemic agent marketed in Japan that has shown glucose-lowering effects in Japanese patients with type 2 diabetes in TIMES trials. However, it is not well known whether imeglimin can affect diabetic kidney disease (DKD). To clarify this, we investigated the potential association between the effects of imeglimin and DKD using diabetic mice (DM) and cultured endothelial cells. Administration of imeglimin significantly lowered urinary albumin excretion in DKD but did not significantly affect blood glucose and glycated albumin. RNA sequencing analysis of the renal cortex indicated the upregulation of antioxidant, oxidative phosphorylation, and AMP-activated protein kinase (AMPK) signaling pathways and downregulation of inflammation, atherosclerosis, and reactive oxygen species. Additionally, imeglimin decreased the mRNA expression of *TNF-α*, *F4/80*, and *fibronectin* in the renal cortex of mice with DKD. In parallel with the findings that imeglimin reduced the protein expression of fibronectin in the renal cortex of mice with DKD, renal histological fibrosis was significantly increased in diabetic mice compared with non-diabetic mice (NDM), which was significantly reduced in mice receiving imeglimin. In an *in vitro* study, high glucose (HG) induced the mRNA expression of *Nlrp3*, *Vcam-1*, and *NOX4*, which were downregulated by imeglimin. These results suggest that imeglimin could improve albuminuria by partially suppressing inflammatory and fibrotic markers in DKD.

## 1 Introduction

Diabetic kidney disease (DKD) is a diabetic vascular complication that can cause end-stage renal failure, which often requires dialysis therapy and renal replacement therapy. DKD is characterized by the appearance of glomerular basement membrane thickening, glomerulosclerosis, podocyte loss, mesangial expansion, and renal fibrosis, clinically leading to the occurrence of albuminuria and the progressive decline in glomerular filtration. In recent years, several RCTs have shown that the addition of SGLT2 inhibitors (Neuen et al., 2019), GLP-1 receptor agonists (GLP-1RAs) (Kristensen et al., 2019), or nonsteroidal mineralocorticoid receptor antagonists (MRAs) (Agarwal et al., 2022) to angiotensin-converting enzyme (ACE) inhibitors or angiotensin receptor blockers (ARBs) can improve the prognosis of DKD. The anti-inflammatory and anti-fibrotic effects of SGLT2 inhibitors, GLP-1RAs, and nonsteroidal MRAs have been considered to treat DKD, but the extent to which they contribute remains unclear (Tuttle et al., 2022; Rayego-Mateos et al., 2023). Moreover, the effectiveness in improving kidney outcomes varies between the clinical trials and is still insufficient to abolish DKD completely. Therefore, it is necessary to elucidate the pathological factors of DKD and to explore additional therapy for intervening residual risks, such as renal inflammation and fibrosis, in the progression of DKD. Especially, a new treatment is needed to inhibit the development of DKD by attenuating the abnormalities of intracellular metabolism and mitochondrial dynamics, increased oxidative stress, and inflammation in DKD.

Imeglimin is an oral hypoglycemic agent marketed in Japan that has shown both glucose-lowering effects and safety in Japanese patients with type 2 diabetes in the 52-week monotherapy trials (Lamb, 2021; Dubourg et al., 2021; Dubourg et al., 2022) (TIMES trial 1, 2), although the frequency of imeglimin treatment in patients with type 2 diabetes was not sufficient. Imeglimin has unique dual effects: it acts on pancreatic β cells and promotes insulin secretion depending on blood glucose concentration (Pacini et al., 2015; Hallakou-Bozec et al., 2021b; Theurey et al., 2022), and it also improves insulin resistance by inhibiting hepatic gluconeogenesis and improving glucose uptake in skeletal muscles. At the cellular and molecular levels, imeglimin may improve mitochondrial dysfunction by partial inhibition of the mitochondrial respiratory chain complex I and correction of complex III activity (Hallakou-Bozec et al., 2021a; Vial et al., 2015). However, it is not well understood whether imeglimin can affect diabetic complications in each organ, nor is the potential association between the effects of imeglimin and DKD well understood (Lachaux M et al., 2020).

In this study, we investigated the potential association between the effects of imeglimin and DKD by using diabetic mice and high glucose (HG)-stimulated cultured endothelial cells.

## 2 Materials and methods

### 2.1 Animals and experimental protocols

Seven-week-old C57BL/6J male mice were purchased from ORIENTAL YEAST CO., LTD. (Tokyo, Japan). The high-fat diet (HFD, #D12492, 60% fat diet) was purchased from Research Diet (Tokyo, Japan). The animals were housed in a temperature-, humidity-, and light-controlled room (12-h light and 12-h dark cycle) and allowed free access to water and a normal chow diet at the Animal Center, Faculty of Medicine, Fukuoka University (Fukuoka, Japan). The animal room was kept at 23 °C during the experiments. Imeglimin was provided by Sumitomo Pharma (Osaka, Japan). C57BL/6 mice (8 weeks old) were fed a control diet (CD) or HFD. For the HFD-fed mice, streptozocin (STZ; 100 mg/kg) was injected intraperitoneally once at 12 weeks of age. STZ-induced diabetic mice were orally administered vehicle or imeglimin (150 mg/kg, twice a day) (Figure 1A) from 16 to 24 weeks of age, resulting in three experimental groups: non-diabetic mice (NDM), diabetic mice (DM), and DM + Imeglimin. Body weight (BW) and casual random blood glucose (BG) were monitored once a week during the experiments. Urinary albumin excretion was measured at 24 weeks of age. At the end of the experiments (24 weeks of age), mice were anesthetized with isopentane, and blood samples from the inferior vein and kidney were stored at −80 °C until use. All experimental protocols were reviewed and approved by the Committee on the Ethics of Animal Experiments, Fukuoka University (Protocol No. 2407028). Mice were randomly assigned to experimental groups. Randomization procedures were applied based on baseline body weight during group allocation, and blinded analysis was employed. All methods involving animals were performed in accordance with the relevant guidelines and regulations.

**FIGURE 1:**
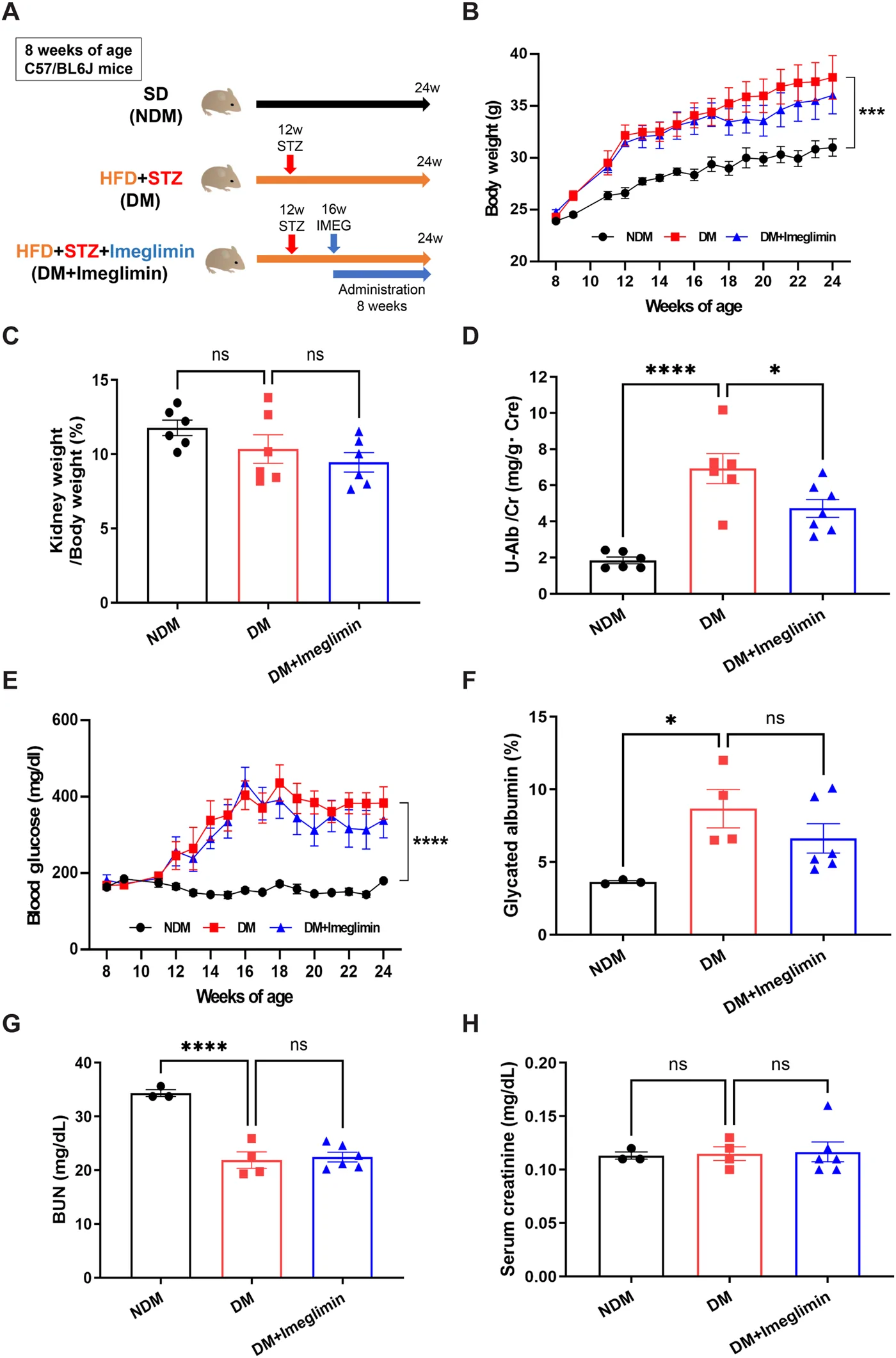
Study design, BW, BG, and urinary albumin excretion from NDM, DM, and DM + Imeglimin. **(A)** Study design. **(B)** Change in BW. **(C)** Kidney weight/body weight ratio. **(D)** Urinary albumin excretion (U-Alb/Cr). **(E)** Change in random BG. **(F)** Glycated albumin. **(G,H)** BUN and serum creatinine. *n* = 3–6 mice/group. Data are expressed as the mean ± SEM. *p < 0.05, **p < 0.01, ***p < 0.001, ****p < 0.0001 (ANOVA).

### 2.2 Chemicals

D-glucose was purchased from Sigma-Aldrich (St. Louis, MO, United States). D-mannitol was purchased from Fujifilm Wako Pure Chemical CO. (Osaka, Japan).

### 2.3 Cell culture

Human umbilical vein endothelial cells (HUVECs) were purchased from LONZA (Basel, Switzerland). HUVECs were seeded into 6-well plates at 2 × 10^4^ cells/well and maintained in EGM^TM^-2 Basal Medium (LONZA) supplemented with EGM^TM^-2 SingleQuots Supplement Pack (LONZA) and 2% fetal bovine serum (FBS). When the cultured cells became 70%–80% confluent, the medium was changed to OPTI-MEM for overnight starvation. D-glucose was added to the cultured medium to induce HG (25 mM). HUVECs were stimulated by HG for 72 h, and vehicle or imeglimin (1 μM or 10 μM) was co-administered.

### 2.4 Quantitative real-time PCR analysis

Total RNA was isolated from HUVECs and renal cortex of mice using NucleoSpin RNA (Macherey-Nagel, Duren, Germany), and cDNA was synthesized as described previously (Otsuka et al., 2022). The mRNA levels were normalized to those of *18S* mRNA. The primer sequences are shown in Table 1.

**TABLE 1:** Primer sequences used in RT-PCR. F = forward, R = reverse.

Gene (mouse)	Primers (5’–3’)	
*18s* (mouse)	F	GCT​TAA​TTT​GAC​TCA​ACA​CGG​GA
	R	AGC​TAT​CAA​TCT​GTC​AAT​CCT​GTC
*Nlrp3* (mouse)	F	CTC​CAA​CCA​TTC​TCT​GAC​CAG
	R	ACA​GAT​TGA​AGT​AAG​GCC​GG
*TNF-α* (mouse)	F	AGG​GTC​TGG​GCC​ATA​GAA​CT
	R	CCA​CCA​CGC​TCT​TCT​GTC​TAC
*F4/80* (mouse)	F	ACC​ACA​ATA​CCT​ACA​TGC​ACC
	R	AAG​CAG​GCG​AGG​AAA​AGA​TAG
*Mcp-1* (mouse)	F	GTC​CCT​GTC​ATG​CTT​CTG​G
	R	GCT​CTC​CAG​CCT​ACT​CAT​TG
*Vcam-1* (mouse)	F	TCT​GAA​CCC​AAA​CAG​AGG​CAG​AGT
	R	AGC​TGG​TAT​CCC​ATC​ACT​TGA​GCA
*Icam-1* (mouse)	F	AGA​TCA​CAT​TCA​CGG​TGC​TG
	R	CTT​CAG​AGG​CAG​GAA​ACA​GG
*Fibronectin* (mouse)	F	GCT​TTG​GCA​GTG​GTC​ATT​TCA​G
	R	ATT​CCC​GAG​GCA​TGT​GCA​G
*TGF-β1* (mouse)	F	GCC​TTT​CCT​GCT​TCT​CAT​GG
	R	TCC​TTG​CGG​AAG​TCA​ATG​TAC
*18s* (human)	F	GTA​ACC​CGT​TGA​ACC​CCA​TT
	R	CCA​TCC​AAT​CGG​TAG​TAG​CG
*Nlrp3* (human)	F	GTG​TTT​CGA​ATC​CCA​CTG​TG
	R	TCT​GCT​TCT​CAC​GTA​CTT​TCT​G
*TNF-α* (human)	F	ACTTTGGAGTGATCGGCC
	R	GCT​TGA​GGG​TTT​GCT​ACA​AC
*Vcam-1* (human)	F	TCT​ACG​CTG​ACA​ATG​AAT​CCT​G
	R	AGG​GCC​ACT​CAA​ATG​AAT​CTC
*Icam-1* (human)	F	CAA​TGT​GCT​ATT​CAA​ACT​GCC​C
	R	CAG​CGT​AGG​GTA​AGG​TTC​TTG
*NOX4* (human)	F	TCA​CAG​AAG​GTT​CCA​AGC​AG
	R	ACT​GAG​AAG​TTG​AGG​GCA​TTC

### 2.5 Western blotting

Fresh mouse renal cortex was homogenized in the RIPA buffer containing protein inhibitor and phosphatase inhibitor (Nacalai Tesque, Kyoto, Japan), prior to SDS-PAGE. Protein bands were visualized using the ECL Western blotting Detection System (Bio-Rad Laboratories, Hercules, CA, United States), and band densities were assessed using ImageJ (NIH) as described previously (Otsuka et al., 2022). Antibodies used for Western blotting are listed in Table 2.

**TABLE 2:** Antibodies used for western blotting.

Primary antibody (target proteins)	Host	Reactivity	MW (kDa)	Providers	Catalog No.	Dilution
Phospho-AMPKα (Thr172) (40H9)	Rabbit	H M R Hm Mk Dm Sc	62	Cell Signaling Technology (Danvers, MA, United States)	2535	1:1000
AMPKα	Rabbit	H M R Mk Hm	62	Cell Signaling Technology (Danvers, MA, United States)	2532	1:1000
Phospho-mTOR (Ser2448)	Rabbit	H M R Mk	289	Cell Signaling Technology (Danvers, MA, United States)	2971	1:1000
mTOR (7C10)	Rabbit	H M R Mk	289	Cell Signaling Technology (Danvers, MA, United States)	2983	1:1000
NLRP3	Rabbit	M R H	118	Abcam (Cambridge, Cambridgeshire, United Kingdom)	ab263899	1:1000
Fibronectin/FN1 (E7F5X)	Rabbit	M	262∼300	Cell Signaling Technology (Danvers, MA, United States)	30903	1:1000
β-Actin	Mouse	​	38–43	Santa Cruz Biotechnology (Dallas, TX, United States)	sc-47778	1:1000

### 2.6 Histological analysis

For evaluation of kidney histology, renal tissue sections were stained with hematoxylin and eosin (H&E), periodic acid–Schiff (PAS), and Masson’s trichrome and observed under a light microscope equipped with a camera. To assess renal fibrosis, Masson’s trichrome-stained images were analyzed using ImageJ software. The images were separated into RGB channels, and the red channel was subtracted from the blue channel to quantify the percentage of fibrotic area relative to the total tissue area.

### 2.7 RNA sequencing analysis

RNA samples were quantified using an ND-1000 spectrophotometer (NanoDrop Technologies, Wilmington, DE), and the quality was confirmed using a TapeStation (Agilent). The sequencing libraries were prepared from 200 ng of total RNA with MGIEasy rRNA Depletion Kit and MGIEasy Fast RNA Library Prep Set (MGI Tech Co., Ltd.) according to the manufacturer’s instructions. The libraries were sequenced on the DNBSEQ-G400 FAST Sequencer (MGI Tech Co., Ltd.) with a paired-end 150 nt strategy. All sequencing reads were trimmed of low-quality bases and adapters with Trimmomatic (v.0.38). Trimmed reads were mapped to the transcript using the Bowtie2 aligner within RSEM. The abundance estimation of genes and isoforms with RSEM generated basic counts data (expected counts). We used the edgeR program to detect the differentially expressed genes (DEGs). Normalized counts per million (CPM) values, log fold-changes (logFC), and *p*-values were obtained from the gene-level raw counts.

Criteria were established for DEGs: *p*-value ≤0.05 for upregulated or downregulated genes for an exploratory basis. The false discovery rate (FDR) criterion was not used for DEG identification. Regarding pathway enrichment statistics, we added the Benjamini–Hochberg procedure and false discovery rate.

### 2.8 Statistical analysis

All data were expressed as the means ± SEM. All relevant datasets were analyzed using one-way ANOVA followed by Tukey’s multiple comparisons test in GraphPad Prism 10 software. A *p*-value <0.05 was considered statistically significant.

## 3 Results

### 3.1 Imeglimin decreases urinary albumin excretion in diabetic mice

To examine the therapeutic effects of imeglimin on DKD progression, we measured BW, BG, and urinary albumin excretion among the three groups. BW was increased in both DM and DM + Imeglimin compared with NDM (*p* < 0.001), while imeglimin did not affect BW in DM and DM + Imeglimin (Figure 1B). At 24 weeks of age, kidney weight/body weight ratio tended to be decreased in DM vs. NDM, which was not significantly affected by imeglimin (Figure 1C). However, urinary albumin excretion was significantly increased in DM compared with NDM (*p* < 0.0001), which was significantly decreased in DM + Imeglimin (*p* < 0.05, Figure 1D). BG was increased in both DM and DM + Imeglimin compared with NDM (*p* < 0.001), but imeglimin did not significantly affect BG between DM and DM + Imeglimin (Figure 1E). Glycated albumin (GA) was significantly increased in DM vs. NDM but was not significantly affected by imeglimin (Figure 1F). BUN was significantly decreased in DM and was not affected by imeglimin (Figure 1G). There were no significant differences in serum creatinine among the three groups (Figure 1H).

### 3.2 RNA sequencing analysis of renal cortex from NDM, DM, and DM + Imeglimin

Given the reduced urinary albumin excretion by imeglimin in DM potentially independent of glucose, we performed transcriptomic profiling of the renal cortex from NDM, DM, and DM + Imeglimin for further investigation. The total number of DEGs is 1,303 (514 upregulated and 789 downregulated genes). Principal component analysis (PCA) of the gene expression pattern revealed marked differences among the groups (Figure 2A). Next, we focused on gene differences in the renal cortices of DM and DM + Imeglimin. A volcano plot with significant differences (|log_2_FC| > 0.58, P < 0.05) in DM + Imeglimin vs. DM revealed that the genes of adrenomedullin (Adm), acyl-CoA synthetase medium-chain family member 3 (Acsm3), and cytochrome P450, family 4, subfamily a, polypeptide 12a (Cyp4a12a), and Hif3a are upregulated, whereas those of leptin, leptin receptor, cxcl9, uncoupling protein 1 (UCP1), TGFβ-induced factor homeobox 2 (Tgif2), activin A receptor, type IC (Acvr1c), cytokine receptor-like factor 1 (Crlf1), tumor necrosis factor receptor superfamily member 19 (Tnfrsf19), Klf5, tumor necrosis factor alpha-induced protein 8-like 3 (Tnfaip8l3), and fibronectin type III domain containing 1 (fndc) are downregulated (Figure 2B).

**FIGURE 2:**
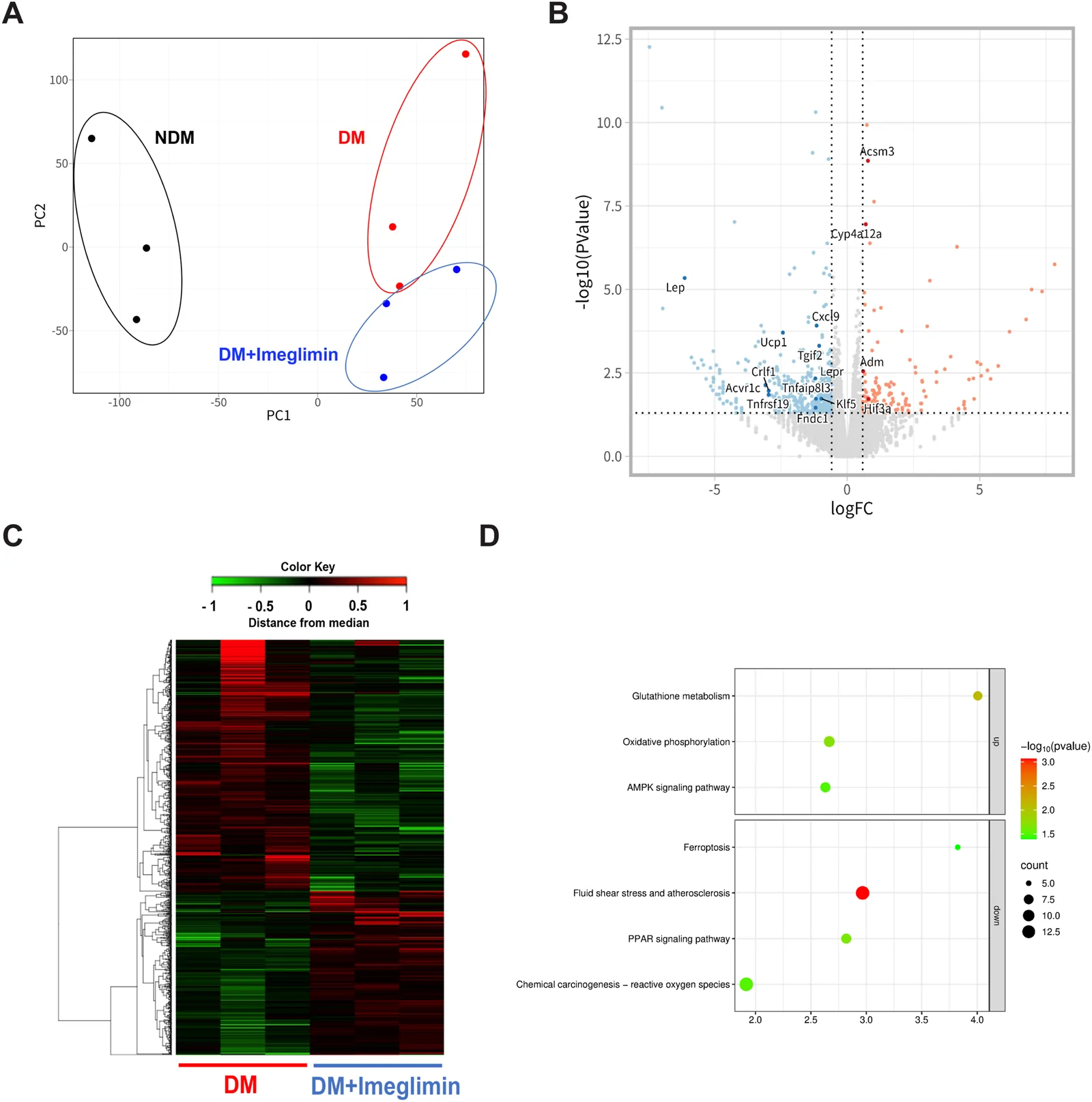
RNA sequencing analysis of the renal cortex from NDM, DM and DM + Imeglimin. **(A)** Principal component analysis (PCA). **(B)** Volcano plot. Upregulated/downregulated genes in DM + Imeglimin vs. DM. *p* < 0.05. |Log_2_FC| > 0.58. **(C)** Heatmap image of the normalized counts in DM and DM + Imeglimin. The color indicates the distance from the median of each row (gene). *p* < 0.05. **(D)** Upregulated and downregulated pathways in DM + Imeglimin vs. DM. *p* < 0.05. *n* = 3 mice/group.

The heatmap, which shows hierarchical clustering using the count data from RNA sequence analysis, revealed quite significant differences in gene expression patterns between DM and DM + Imeglimin (*p* < 0.05, Figure 2C). Kyoto Encyclopedia of Genes and Genomes (KEGG) pathway analysis revealed significant enrichment in several pathways in the renal cortex from DM + Imeglimin vs. DM. The upregulated pathways in DM + Imeglimin are associated with glutathione metabolism, oxidative phosphorylation, and the AMPK (AMP-activated protein kinase) signaling pathway, while downregulated pathways are associated with ferroptosis, fluid shear and atherosclerosis, PPAR signaling pathway, and chemical carcinogenesis-reactive oxygen species (*p* < 0.05, Figures 2D,E). We verified the consistency of the KEGG pathway enrichment analysis with the results from the Benjamini–Hochberg procedure and FDR in Table 3.

**TABLE 3:** KEGG pathway enrichment analysis for the RNA-seq dataset.

Term	p-value	Benjamini–Hochberg	FDR
(a) Upregulated			
mmu00480: Glutathione metabolism	0.007738	0.310621765	0.304429969
mmu00190: Oxidative phosphorylation	0.019446	0.450238681	0.441263823
mmu04152: AMPK signaling pathway	0.032063	0.603181816	0.591158258

Term	p-value	Benjamini–Hochberg	FDR
(b) Downregulated			
mmu05418: Fluid shear stress and atherosclerosis	8.50E-04	0.016656907	0.015807065
mmu03320: PPAR signaling pathway	0.022885	0.305820382	0.290217301
mmu05208: Chemical carcinogenesis, reactive oxygen species	0.030874	0.363083326	0.344558666
mmu04216: Ferroptosis	0.0403	0.423146556	0.401557446

FDR, false discovery rate.

### 3.3 Imeglimin attenuates the gene expression of inflammatory cytokines and fibronectin in the renal cortex of diabetic mice

Next, based on the RNA sequencing analysis of the renal cortex from DM and DM + Imeglimin, we focused on the genes downregulated by imeglimin, especially related to TGF-β, tumor necrosis factor (TNF), and fibronectin. To ask whether imeglimin inhibits inflammation and fibrosis in DKD progression, real-time qPCR was performed to evaluate the expression of inflammation- and fibrosis-related genes in the renal cortex from NDM, DM, and DM + Imeglimin. The mRNA expressions of *Nlrp3*, *TNF-*α, *F4/80*, *Mcp-1*, *Vcam-1*, *Icam-1*, and *fibronectin* were significantly increased in DM compared with NDM (*p* < 0.05, 0.01, 0.01, 0.01, 0.05, 0.05, and 0.01 vs. NDM, respectively, Figures 3A–G), whereas imeglimin decreased the elevated mRNA expressions of *TNF-*α, *F4/80,* and *fibronectin* (*p* < 0.05, 0.05, and 0.01 vs. DM, respectively, Figures 3B,C,G). In addition, imeglimin tended to reduce the elevated mRNA expressions of *Nlrp3*, *Mcp-1*, *Vcam-1*, *Icam-1*, and *TGF-β* in DM, although there were no statistically significant differences (Figures 3A,D–F,H).

**FIGURE 3:**
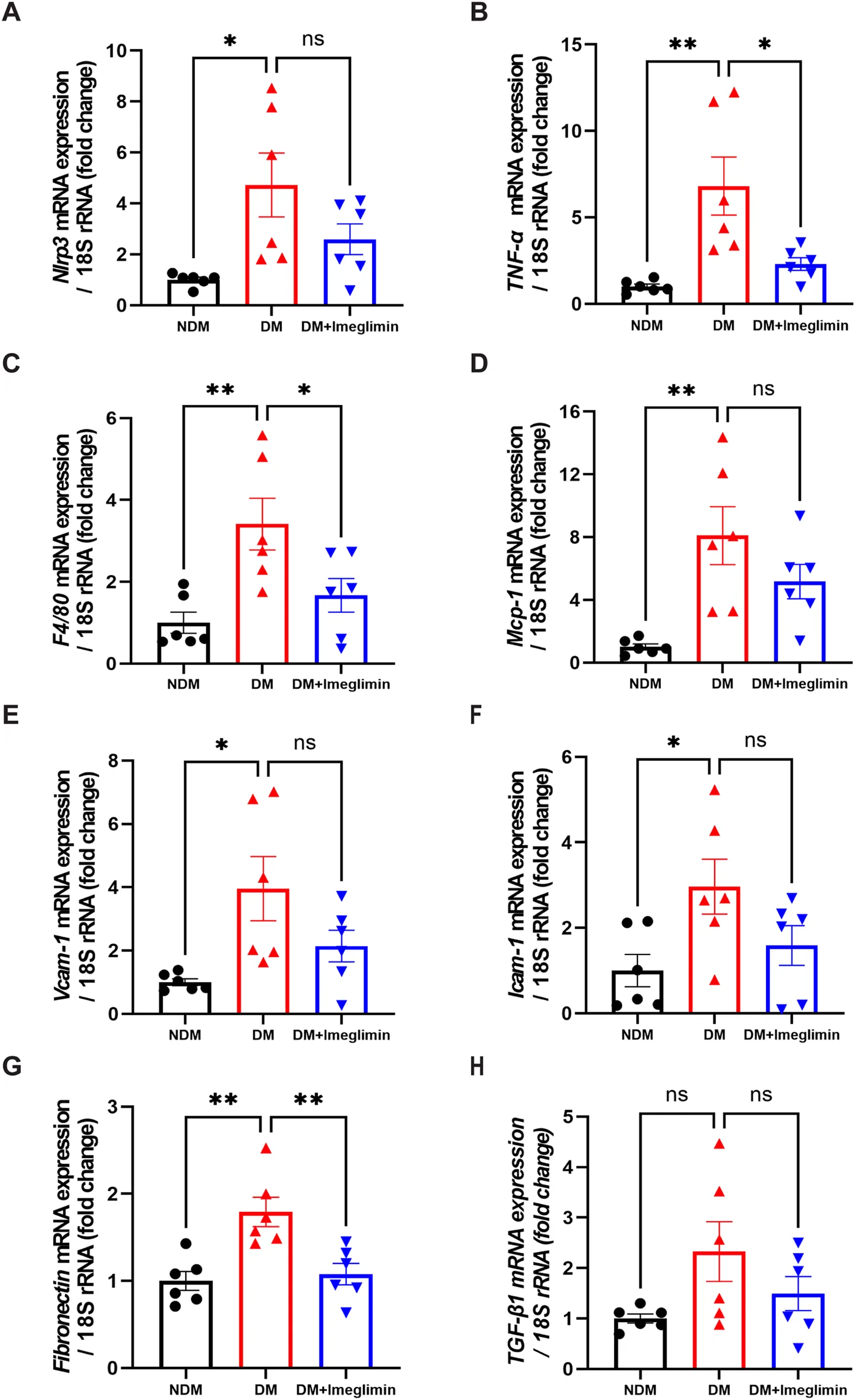
Gene expression levels in the renal cortex from NDM, DM and DM + Imeglimin. The mRNA expression levels related to inflammation, including *Nlrp3*, *TNF-*α, *F4/80*, and *Mcp-1* **(A-D)**, those related to cell adhesion, including *Vcam-1* and *Icam-1* **(E, F)**, and those related to fibrosis, including *fibronectin* **(G)** and *TGF-β1* **(H)**, were quantified by real-time PCR. *n* = 6 mice/group. Data were expressed as the mean ± SEM. ^✱^ *p* < 0.05, ^✱✱^ *p* < 0.01 (ANOVA). ns, not significant.

### 3.4 Imeglimin attenuates protein expression of fibronectin in the renal cortex of diabetic mice

Based on the pathway analysis from RNA sequencing of renal cortices from DM and DM + Imeglimin (Figure 2D), we asked whether imeglimin can affect the protein expression of AMPK and mTOR signaling pathway, both of which play critical roles in the development of DKD. There were no differences in phosphorylation of AMPK and mTOR between DM and DM + Imeglimin (Figures 4A–C). Because imeglimin administration was associated with reduced mRNA expression of inflammatory markers and fibronectin in the renal cortex of DM, we next examined the protein levels by Western blotting. Imeglimin tended to reduce the elevated protein expression of NLRP3 in DM, although there were no statistically significant differences (Figures 4A,D). Interestingly, the protein expression of fibronectin was significantly increased in DM compared with NDM (*p* < 0.001), which was significantly decreased by imeglimin (*p* < 0.05, Figures 4A,E).

**FIGURE 4:**
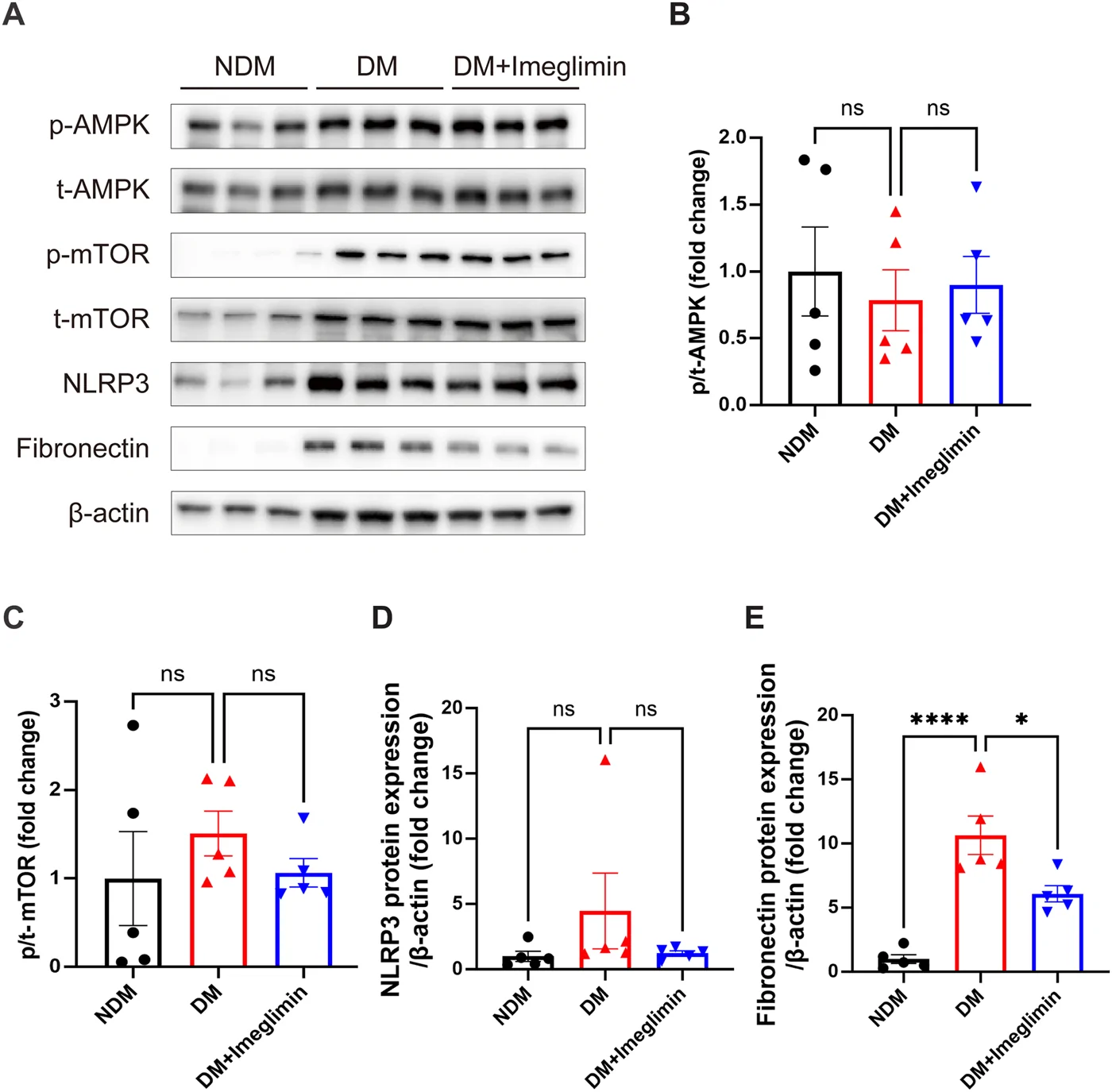
Protein expression levels of the phosphorylated AMPK (p-AMPK), phosphorylated mTOR (p-mTOR), NLRP3, and fibronectin in the renal cortex from NDM, DM, and DM + Imeglimin. **(A)** Representative Western blots for the assessment of p-AMPK, t-AMPK, p-mTOR, t-mTOR, NLRP3, fibronectin, and β-actin. **(B–E)** Quantitative bar graphs. *n* = 5 mice/group. Data are expressed as the mean ± SEM. *p < 0.05, **p < 0.0001 (ANOVA).

### 3.5 Imeglimin reduced renal fibrosis in diabetic mice

Histological assessment of the renal cortex from NDM, DM, and DM + Imeglimin was performed by H&E, PAS, and Masson’s trichrome staining (Figure 5A). H&E and PAS staining showed no apparent diabetic glomerular lesions, such as glomerular basement membrane thickening or mesangial matrix expansion, in either DM or DM + Imeglimin compared with NDM. Next, quantitative pathological assessment for renal fibrosis was performed using Masson’s trichrome staining. In parallel with the findings that imeglimin reduced the protein expression of fibronectin in the renal cortex of DKD (Figure 4E), renal fibrosis was significantly increased in DM compared with NDM (*p* < 0.01), and it was significantly reduced by imeglimin (*p* < 0.05) (Figure 5B).

**FIGURE 5:**
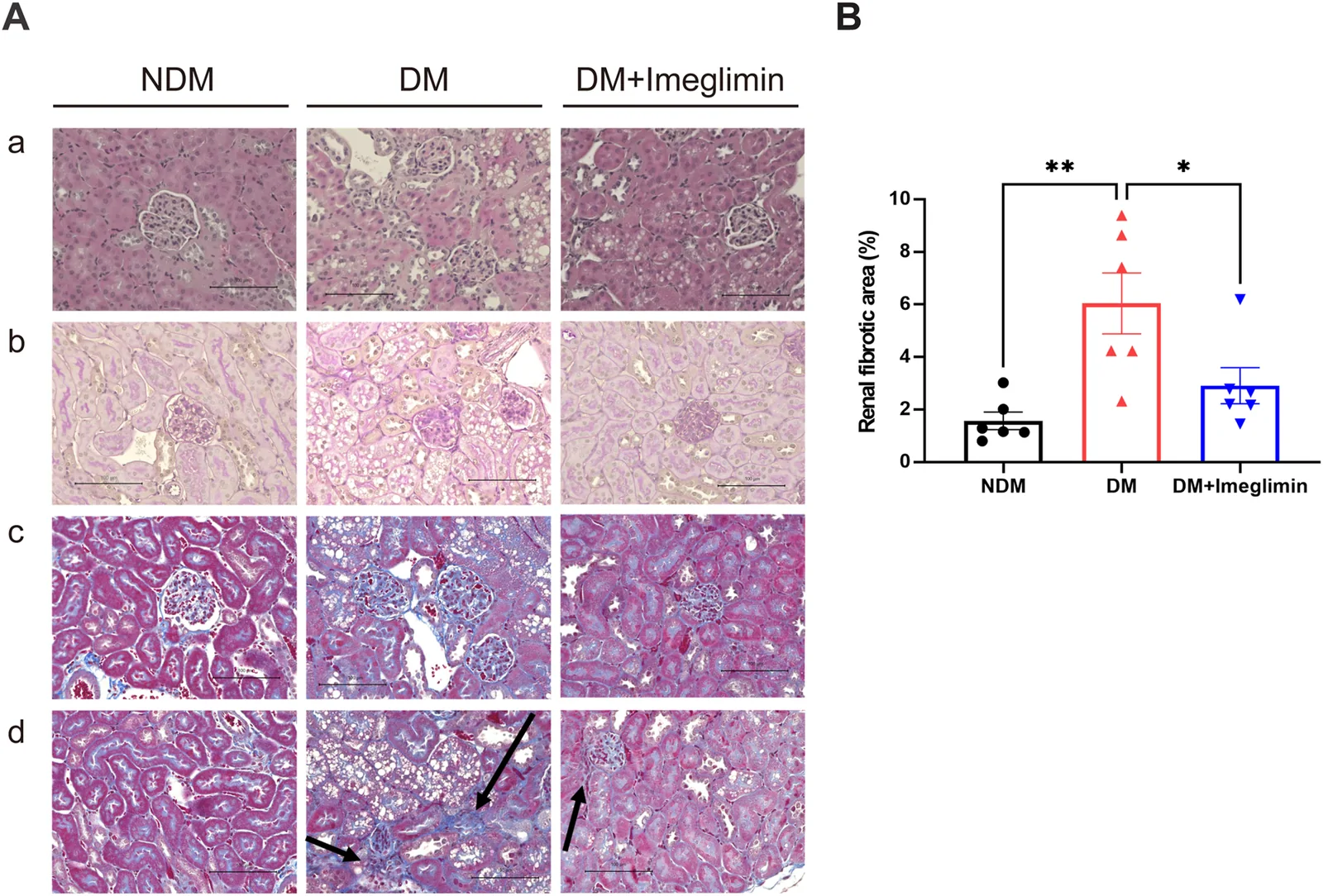
Histological assessment of renal cortices from NDM, DM and DM + Imeglimin. **(A)** Representative images for renal tissue sections stained with **(a)** hematoxylin and eosin **(H&E)**, **(b)** periodic acid–Schiff (PAS), and **(C,D)** Masson’s trichrome. **(c, d)** Images focused on the glomeruli and renal interstitium, respectively. Renal interstitial fibrosis was observed in DM and DM + Imeglimin (solid arrow). Scale bar, 100 μm. Original magnification, ×400. **(B)** Renal fibrotic score on the Masson trichrome staining. *n* = 6 mice/group. Data are expressed as the mean ± SEM. *p < 0.05, **p < 0.01 (ANOVA).

### 3.6 Imeglimin reduced gene expressions of Nlrp3, Vcam-1, and NOX4 in HG-stimulated HUVECs

Based on the pathway analysis from RNA sequencing of the renal cortex from DM and DM + Imeglimin (Figure 2D), we asked whether imeglimin can prevent the progression of DM-induced endothelial dysfunction, such as inflammation and oxidative stress. To answer this, we examined the effects of imeglimin on the mRNA expression of *Nlrp3*, *TNF-α*, *Vcam-1*, *Icam-1*, and *NOX4* under HG stimulation in cultured HUVECs. The mRNA expression of *Nlrp3* was increased by 25 mM HG, but not 25 mM mannitol, compared with low glucose (LG, 5 mM), which was significantly downregulated by both 1 μM and 10 μM imeglimin (*p* < 0.05 and 0.01 vs. HG, respectively, Figure 6A). Imeglimin tended to reduce the elevated mRNA expression of *TNF-α* in HG, although there were no statistically significant differences (Figure 6B). The mRNA expression of adhesion molecule *Vcam-1* was increased by HG compared with LG, which was significantly decreased by 10 μM imeglimin (*p* < 0.05 vs. HG, Figure 6C), while that of *Icam-1* was not different among LG, HG, and HG + Imeglimin (Figure 6D). On the other hand, the mRNA expression of *NOX4* was increased by HG compared with LG, while the expression of *NOX4* was significantly decreased by both 1 μM and 10 μM imeglimin (both *p* < 0.001 vs. HG, Figure 6E).

**FIGURE 6:**
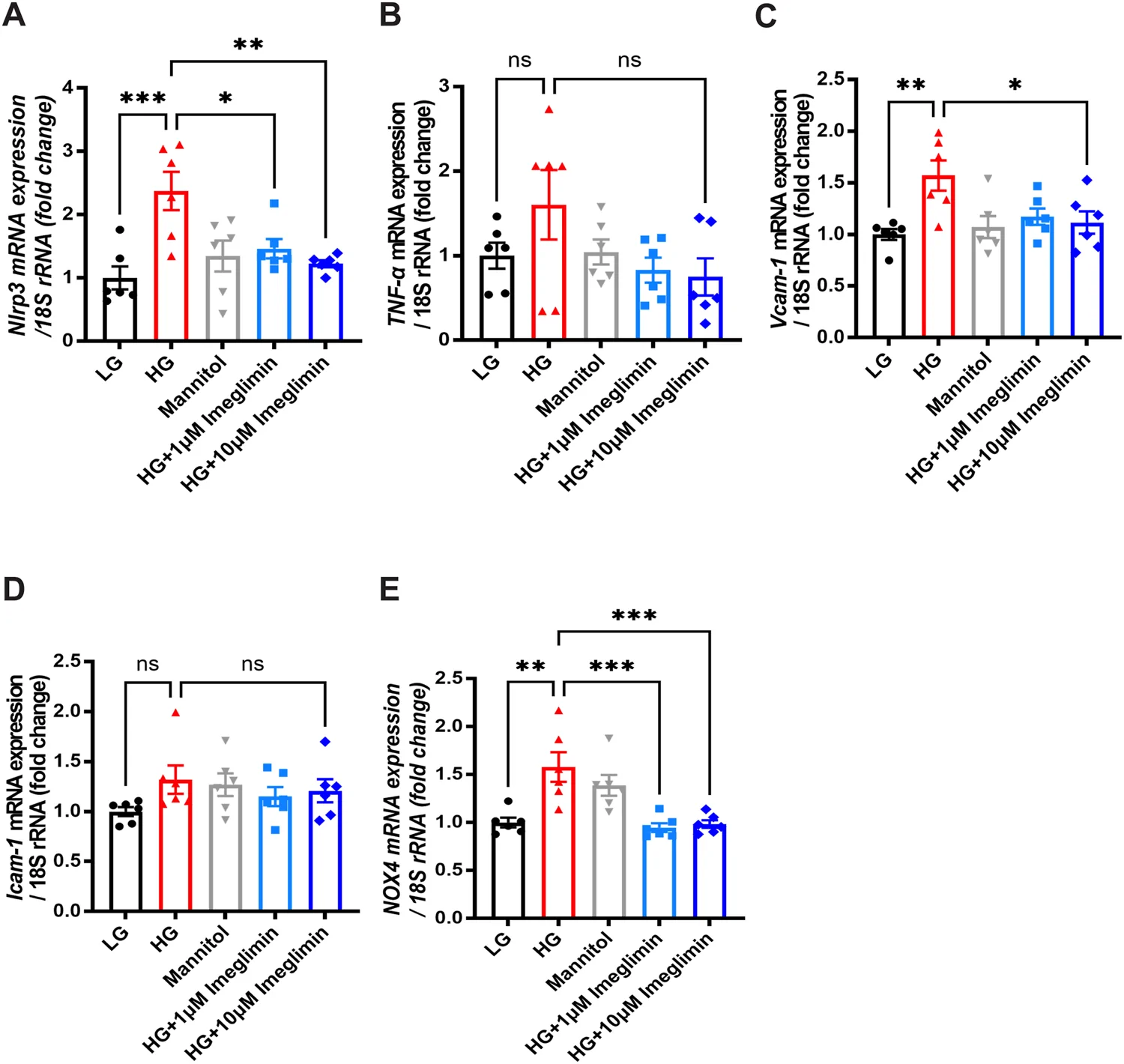
Gene expression levels in LG, HG, and HG + Imeglimin in HUVECs. The mRNA expression levels related to inflammation, including *Nlrp3* **(A)** and *TNF-α* **(B)**, those related to cell adhesion, including *Vcam-1* and *Icam-1* **(C,D)**, and those related to oxidative stress, including NADPH oxidase 4 (NOX4) **(E)**, are quantified by real-time PCR. *n* = 6/group. Data were compared as the mean ± SEM. *p < 0.05, **p < 0.01, ***p < 0.001 (ANOVA). ns, not significant.

## 4 Discussion

The increasing number of patients with DKD is a global social concern (Ogurtsova et al., 2017). A “STOP-DKD declaration” adopted by the Japan Diabetes Society and the Japanese Society of Nephrology in 2017 highlighted that it would be meaningful, especially for those patients with DKD, to elucidate the pathophysiology of DKD and the residual risk of DKD, and to develop novel therapy for DKD (Zullig et al., 2020; Machen et al., 2022). In this research, we demonstrate that imeglimin may have a potential association with DKD through its actions on diabetes- or high glucose-induced intracellular abnormalities, including cellular inflammation, cell adhesion, fibrosis, and oxidative stress (Brownlee, 2005).

Previous studies have shown that administration of imeglimin improves renal fibrosis and decreases urinary albumin excretion in Zucker *fa/fa* rats as well as reduces arterial atherosclerosis in diabetic mice (Sanada et al., 2024). However, the molecular mechanisms by which imeglimin protects against DKD and atherosclerosis remained poorly understood. Here, our study demonstrated that imeglimin significantly reduced urinary albumin excretion in diabetic mice. Although imeglimin is known to exert blood glucose-lowering effects (Song et al., 2025), its underlying mechanism may involve improvement of mitochondrial dysfunction, which may be partly independent of glucose control in multiple cell types such as pancreatic β cells and liver cells (Vial et al., 2015; Li et al., 2022; Vial et al., 2021; Li et al., 2026; Kaji et al., 2024). Previous studies have suggested that imeglimin may improve mitochondrial function, possibly by partial inhibition of the mitochondrial respiratory chain complex I and enhanced activity of complex III (Li et al., 2024; Kato et al., 2025). Interestingly, because a glucose-lowering effect of imeglimin was not significantly observed between DM and DM + Imeglimin in our study, it is possible that the effect of imeglimin on reduced albuminuria is potentially independent of blood glucose levels, suggesting its additional association with DKD.

Recently, inflammation and fibrosis of renal tissue have been identified as possible new therapeutic targets to prevent the progression of DKD (Mora-Fernández et al., 2014; Alicic et al., 2017; Niewczas et al., 2019). NLRP3 inflammasome is known as a key factor of the inflammatory response related to DKD (Mangan et al., 2018; Tang and Yiu, 2020), and its activation mediates inflammatory cytokine secretion such as IL-18 and IL-1β (Qiu and Tang, 2016; Liu et al., 2021). In an *in vivo* study, imeglimin administration decreased the mRNA expression of *F4/80*, known as a mature macrophage marker, and the inflammatory cytokine *TNF-α*. Although pathway analysis from RNA sequencing showed imeglimin may upregulate the AMPK signaling pathway in DKD, imeglimin tended to increase the phosphorylation levels of AMPK, although not significantly. Similarly, imeglimin did not significantly decrease the phosphorylation level of mTOR and the protein expression of NLRP3 in the renal cortex of diabetic mice. Therefore, these pathways may not fully explain the observed effects of imeglimin.

Regarding the fibrosis of renal tissue in diabetic mice, imeglimin decreased the mRNA expression of *fibronectin,* which plays a main role in the accumulation of extracellular matrix in DKD. Additionally, imeglimin reduced the protein expression of fibronectin in the renal cortex of diabetic mice. In parallel, renal fibrotic histological changes were reduced by imeglimin in DKD. Taken together, our findings suggest that imeglimin is associated with partial suppression of inflammatory and fibrotic markers in DKD, which may lead to reduced urinary albumin excretion. Additional mechanistic experiments are needed to clarify the renoprotective actions of imeglimin.

Numerous studies have indicated that damage to glomerular endothelial cells (ECs) is critical in the progression of DKD (Mohandes et al., 2023; Chen et al., 2023). Recently, imeglimin has been reported to improve vascular endothelial function in patients with type 2 diabetes (Uchida et al., 2023). In this study using cultured HUVECs, imeglimin administration reduced HG-induced *Nlrp3* and *Vcam-1* gene expressions. Additionally, the gene expression of *NOX4*, an oxidative stress marker in kidney tissue, was also reduced by imeglimin administration. These results indicated that not only anti-inflammatory and anti-fibrotic but also antioxidant stress actions of imeglimin on vascular EC may be partly associated with DKD. However, because renal tissue ROS levels or oxidative damage markers such as MDA and 8-OHdG were not measured in this study, further experiments are needed to investigate whether imeglimin may have antioxidant stress action in DKD.

Taken together, our findings suggest that the effects of imeglimin on DKD are not only limited to its blood glucose-lowering effect and modulation of mitochondrial function but also have a potential association with anti-inflammatory, anti-fibrotic, and anti-oxidant actions in renal tissue (Figure 7). Because a recent study has reported the long-term safety and sustained glycemic efficacy of imeglimin in Japanese patients with type 2 diabetes and CKD stage G3b-5 by dose adjustment (TWINKLE) (Babazono et al., 2025), further investigation will be required to clarify how imeglimin protects against DKD progression by both direct and indirect actions.

**FIGURE 7:**
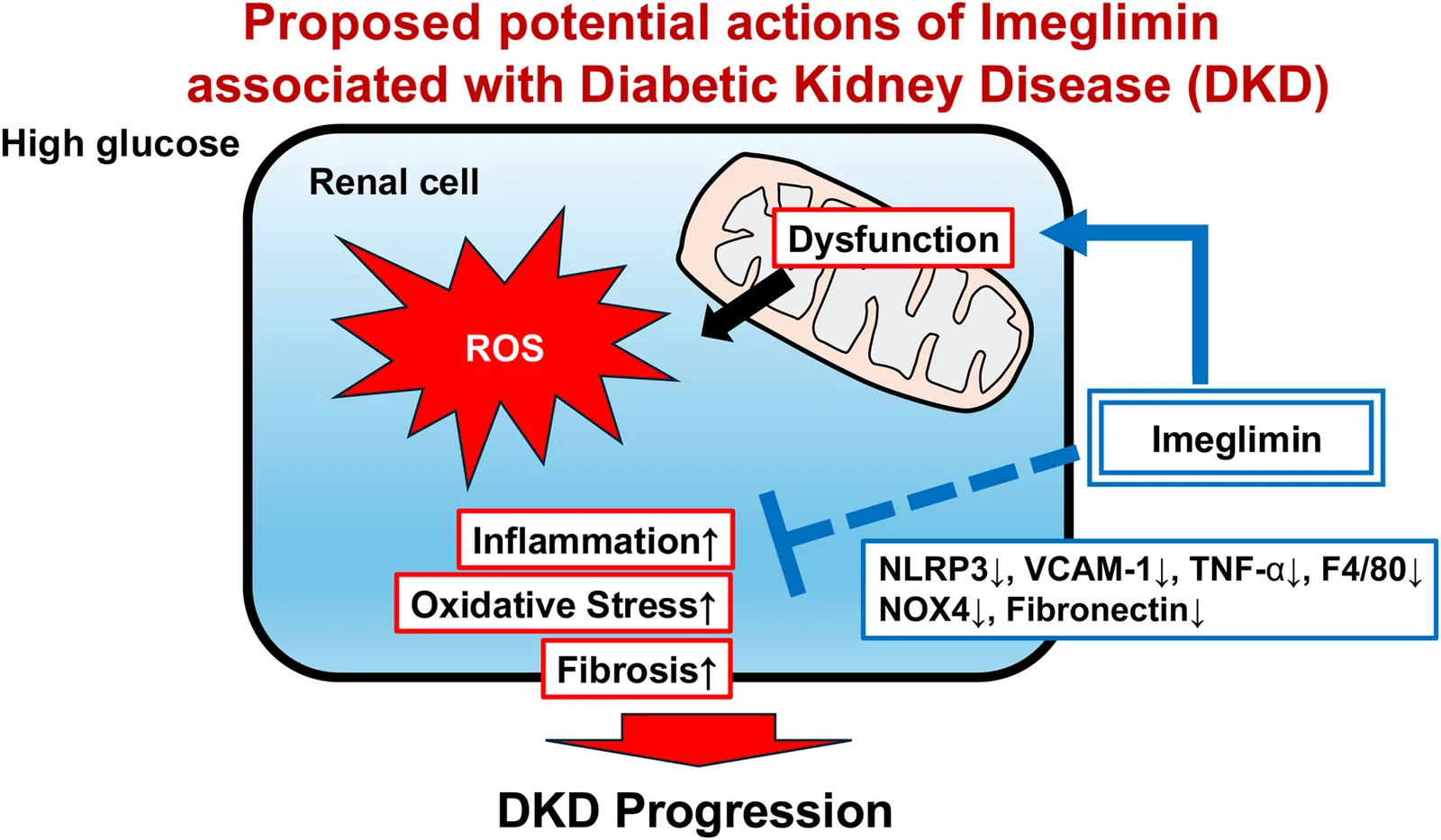
Proposed potential actions of imeglimin associated with DKD from this study. Dashed lines indicate trending changes that did not reach statistical significance.

There are some limitations of this study. (1) Imeglimin did not affect random BG and GA between DM and DM + Imeglimin, which is partly consistent with a previous report (Nihei et al., 2026). Because HbA1c was not measured and an oral glucose tolerance test or repeated-measures statistical methods in BG were not performed, we cannot completely exclude the possibility that imeglimin may affect glycemic control. (2) The *in vivo* dose of 150 mg/kg twice daily and the *in vitro* doses of 1 μM and 10 μM imeglimin were determined by previous reports (Vial et al., 2021; Nihei et al., 2026; Awazawa et al., 2024). Because the concentration of imeglimin in the plasma was not measured in this study, the translational relevance from mice and cultured cells to humans will need to be supported by pharmacokinetic assessment. (3) The cellular experiments using HUVECs exposed to high glucose provided some supportive evidence on imeglimin. However, because HUVECs are not kidney-specific endothelial cells, the translational relevance to renal endothelial cells should be interpreted with caution. In addition, cellular experiments primarily relied on mRNA expression without protein-level validation or functional assays such as ROS production, monocyte adhesion, or endothelial permeability. Therefore, the potential antioxidant effects of imeglimin are still speculative and need further investigation. (4) The sample size in each experiment was relatively small, and formal power calculations were not performed in this study. (5) For RNA-seq analysis, DEGs were identified using a nominal *p*-value <0.05 without FDR correction, indicating that the RNA-seq data should be considered hypothesis-generating rather than mechanistic evidence. (6) Imeglimin did not significantly affect the phosphorylation levels of AMPK and mTOR, as well as NLRP3 expression, indicating that these related pathways may not explain the observed effects and the proposed mechanism remains speculative. (7) The clinical evidence for protective effects of Imeglimin against DKD is not well established. Therefore, the lack of human data represents a limitation of the present study. Clinical studies are required to fully evaluate the efficacy of imeglimin in patients with DKD.

In conclusion, imeglimin may improve albuminuria and partially suppress inflammatory and fibrotic markers in DKD. Additional mechanistic experiments are needed to investigate the renoprotective roles of imeglimin.

## Data Availability

The data presented in the study are deposited in the GEO repository, accession number GSE344280.
